# Letter to the editor: the global summit on the efficacy and effectiveness of spinal manipulative therapy for the prevention and treatment of non-musculoskeletal disorders: a systematic review of the literature

**DOI:** 10.1186/s12998-021-00378-1

**Published:** 2021-07-20

**Authors:** Dana J. Lawrence

**Affiliations:** Parker University, Dallas, TX USA

To the editor:

There will be many reasons to criticize and critique the report from the Global Summit on the Efficacy of Spinal Manipulative Therapy [1], and I am certain that this paper will receive its fair share of such criticism. I will let others do that and prefer here to focus on a few smaller and perhaps more subtle issues I see while reading the paper.

The authors are clear where they make assumptions. Unfortunately, these assumptions are simply that, since they are not supported by actual data. For example, in discussing limitations, they note that the critical appraisal of the papers in this review might vary among the various and many reviewers. This is a legitimate concern, but it is one that is brushed away by then stating that the 4-step process used “likely” minimized the problem. Did it? Can this be demonstrated? And again, the authors state that they do not believe publication bias is present because studies most unlikely to be published are those that failed to obtain a positive result. While this may in fact be true for other disciplines, there is no proof offered that it is true within chiropractic or the fields covering spinal manipulation. It is true that in the pharmaceutical world publication bias exists because of failure of drug companies to publish negative results and of course we do have evidence that editors prefer significant findings. This is not in dispute. However, publication bias tilted toward positive, not negative, findings has been demonstrated even in Cochrane reviews themselves [2]. Again, though we know that editors do favor papers with significant (positive) findings, there are other reasons papers may not be published, including failure to accrue, departure of researchers or research teams and so on. Those papers may have positive or negative results. One cannot assume, and if I did I could also assume that editors in traditional biomedicine have a preference toward rejecting findings with positive results that favor chiropractic over those that do not. My own paper demonstrated that bias may exist in our field, but we did not determine in what direction results might have been affected [3]. These assumptions made by the authors have not been tested here.

While this may seem a minor point in the paper, I do not think it is. It represents a set of assumptions made by the authors that I do not feel can be supported and thus call into question potential bias. Limitations that may exist and that may impact results are being rejected without factual support. In a paper that calls into question the professional practices of literally thousands of practicing DCs, none of whom had a voice in this project, we need to do better.

Sincerely,

Dana J. Lawrence, DC, MMedEd, MA.

Associate Provost of Education and Research.

Parker University.

Dallas, TX USA.

## Data Availability

Not applicable.
